# Clinical efficacy and safety of secukinumab in the treatment of generalized pustular psoriasis in the pediatric population: a systematic review of the literature

**DOI:** 10.3389/fmed.2024.1377381

**Published:** 2024-08-09

**Authors:** Kebo Wei, Ping Li, Xin He, Dongyue Yang, Jing Lang, Lingyao Lai, Min Xiao

**Affiliations:** ^1^Clinical Research on Skin Diseases School of Clinical Medicine, Chengdu University of TCM, Chengdu, China; ^2^Dermatology of Department, Hospital of Chengdu University of Traditional Chinese Medicine, Chengdu, China

**Keywords:** secukinumab, generalized pustular psoriasis, systematic review, pediatric, efficacy and safety

## Abstract

**Background:**

Generalized pustular psoriasis (GPP) is a severe type of psoriasis. The current treatment primarily relies on corticosteroids and immunosuppressants. In recent years, biologics have been increasingly utilized in the treatment of this disease, and have demonstrated good clinical efficacy. However, children and adolescents are primarily treated with immunosuppressants, which have limited clinical application due to the serious side effects they may cause. At the same time, the effectiveness of current treatments is unsatisfactory. Secukinumab has been widely reported to be effective and safe in treating this disease. However, there are still insufficient data on its use in treating GPP in children.

**Objective:**

To conduct a systematic review of the existing literature on the use of secukinumab for treating generalized pustular psoriasis in children and adolescents, and to evaluate its clinical effectiveness and safety.

**Methods:**

We conducted a systematic review of all the literature reporting on the treatment of GPP in children and adolescents with secukinumab.

**Results:**

A total of 7 papers (46 patients) were included in this study. After 12 weeks of treatment, all 46 participants were able to achieve a GPPASI score of 90 or higher, with approximately 96% of patients achieving complete clearing of the lesions (GPPASI 100 or JDA0). Adverse events were reported in 8 patients, the rate of adverse reactions was approximately 17%.

**Conclusion:**

The treatment of GPP in children and adolescents with secukinumab has a rapid onset of action and a high safety profile. However, the results of the literature may be influenced by publication bias.

## 1 Introduction

Generalized pustular psoriasis is a relatively rare and severe immunoinflammatory skin disease characterized by recurrent episodes of widespread, noninfectious, visible pustules and erythema, which can be severely burdensome and even life-threatening (1, 2). Acute generalized pustular psoriasis is often linked to a severe systemic inflammatory response, including fever, elevated white blood cell count, and abnormal liver function (3–5). The disease can be clinically categorized into several subtypes, including acute GPP, pustular psoriasis of pregnancy, pustular psoriasis annularis, and infantile/adolescent pustular psoriasis (6). There are wide regional variations in the prevalence of the disease. A retrospective study in France reported a rate of about 1.4 cases per million people (7), while a study in Korea reported a range of 88–124 patients per million people (8). Current studies suggest that the interleukin-36 (IL-36) cytokine signaling pathway plays a key role in the development of this disease (9, 10). Mutations in the IL36RN gene have been associated with severe GPP, which is characterized by an early onset of the disease, more systemic inflammation, lack of associated plaque psoriasis, and dependence on systemic therapy (11, 12). The current treatment of the disease primarily relies on immunosuppressive agents, such as cyclosporine, acitretin, and methotrexate (13). However, much of the rationale for these treatments is derived from the management of plaque psoriasis. There is a shortage of high-quality multicenter clinical evidence for the use of these drugs in treating GPP, and even less evidence for their use in treating pustular psoriasis in children and adolescents.

As research on this disease intensifies, an increasing number of biological agents are being used to treat GPP, such as adalimumab, secukinumab, guselkumab, and others (14). In response to the significant role of the IL-36 signaling pathway in GPP, Spesolimab has been utilized in the United States and Europe to treat adult GPP, demonstrating favorable efficacy (15). However, treating GPP in children and adolescents remains a current therapeutic challenge. Immunosuppressants are currently the first-line treatment options, but their effectiveness often fails to satisfy patients. The search for a highly effective and safe treatment option for pediatric and adolescent patients is a current clinical priority. Secukinumab is a fully human monoclonal antibody that targets IL-17A, specifically binding to and neutralizing its biological activity. This action inhibits inflammatory cytokine and chemokine networks (16). Currently, secukinumab has achieved a favorable safety profile in the treatment of plaque psoriasis in children and adolescents (17). The aim of this review was to systematically evaluate the literature on the use of secukinumab for the treatment of GPP in children and adolescents.

## 2 Methods

The systematic review was conducted and reported in accordance with the Preferred Reporting Items for Systematic Reviews and Meta-Analyses (PRISMA) statement (18). We searched some databases until in January 2024, including PubMed, Embase, Web of Science, and the Cochrane Library. We searched PubMed using the following keywords: “Generalized pustular psoriasis,” “children,” “adolescents,” and “Secukinumab.”

### 2.1 Eligibility criteria

We included all studies, such as randomized controlled trials (RCTs), retrospective studies, and case reports, that focused on the treatment of pustular psoriasis in children and adolescents with generalized pustular psoriasis treated with Secukinumab.

### 2.2 Indicators

The primary indicators mainly included the GPP lesion area and severity index (GPPASI) (19). Complete remission was defined as GPPASI100, while GPPASI 50 indicated half of clinical remission, and so on. The secondary indicators showed adverse reactions.

### 2.3 Study selection and data extraction

Two independent authors, Kebo Wei and Xin He, screened the titles and abstracts of studies based on the eligibility criteria and then excluded irrelevant studies. The full text of the remaining studies was then reviewed to identify those for inclusion. The first author extracted fundamental data from the included literature, such as age, gender, previous treatments, treatment durations, and efficacy indicators, and documented them in tables for statistical analysis. The publications were also categorized based on the evidence register: (A) prospective studies, (B) retrospective studies, and (C) case studies or case report series.

### 2.4 Statistical analysis

Data were analyzed using descriptive statistics. Categorical variables are presented as number (%), and continuous variables are presented as mean ± standard deviation or median (range).

## 3 Results

**Figure 1:**
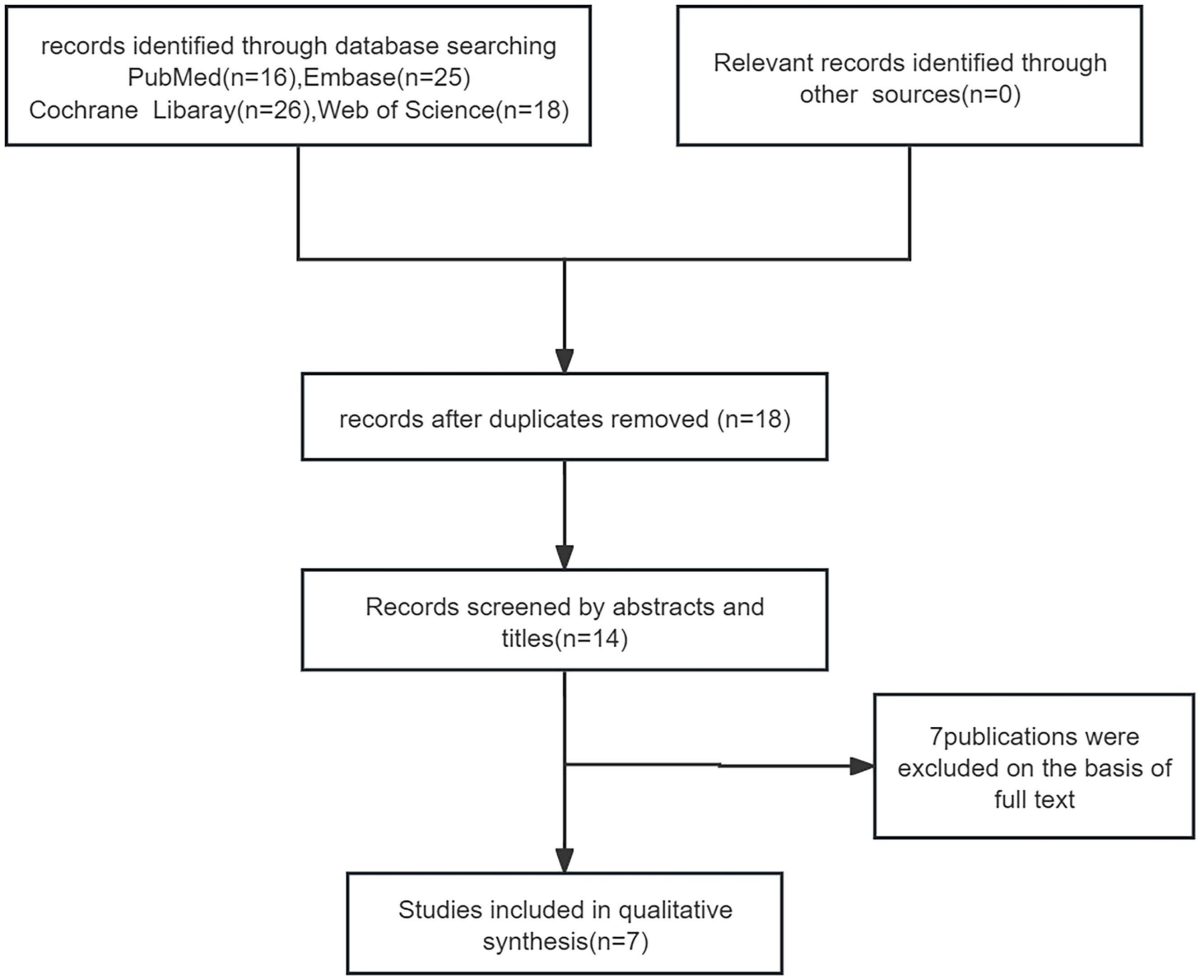
Selection of the included studies.

**Figure 2:**
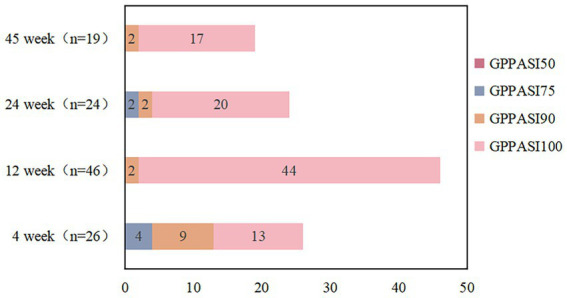
GPPASI scores at different treatment times.

We reviewed 85 papers and ultimately included 7 papers in the final study (Figure 1), which encompassed a total of 46 patients. Among these, 1 was a retrospective study, 1 was a randomized controlled study, and the remaining were case reports (20–26). The average age of the 46 patients included in the literature was 8.23 ± 2.6 years old, and 16 (35%) were female. Of the 46 patients, only 25 reported previous treatments. Among the 25 reported patients, 5 had been treated with methotrexate, 17 with acitretin, 2 with cyclosporine, 4 with oral corticosteroids, 1 with etanercept and adalimumab, and 1 with AnaKinra (Table 1). In terms of efficacy (Figure 2), at 4 weeks after secukinumab initiation, 26 patients reported clinical outcomes, all 26 patients reported achieving a GPPASI of 75 or higher, with 13 (50%) reaching a GPPASI of 100, and 9 (35%) reaching a GPPASI of 90. At 12 weeks, 46 patients had achieved a GPPASI of 90, with 44 (96%) of these patients reaching a GPPASI score of 100, indicating good efficacy. At 24 weeks, 24 patients reported clinical outcomes. Out of the 24 patients reported, all patients achieved a GPPASI of 75 or higher, with 22 (92%) reaching a GPPASI of 90 and 20 (83%) reaching a GPPASI of 100. At 45 weeks, efficacy was reported for a total of 19 patients, with all patients achieving a GPPASI of 90 and 17 (89%) maintaining a GPPASI of 100. In summary, the results showed that all patients achieved at least 90% clinical remission at 12 weeks. By 45 weeks, out of the 19 patients reported the outcomes, all patients achieved at least 75% remission, demonstrating the significant efficacy of secukinumab in both short-term and long-term treatment of children and adolescents. In terms of safety, adverse events were reported in 8 (17%) patients. Among these, 2 patients experienced elevated levels of alanine aminotransferase, 2 patients developed atopic dermatitis-like lesions, 2 patients had mild neutropenia, 1 patient had herpes simplex, and 1 patient had a respiratory infection. There were no serious adverse events, indicating a good safety profile.

**Table 1:** Characteristics of the included studies.

Baseline characteristics	*n* (*N*)
Patients (*N*)	46
Age, mean ± (SD)y	8.23 ± 2.6
Female	16 (46)
Adverse effects	8 (46)
The elevated level of alanine aminotransferase	2 (46)
Atopic dermatitislike lesions	2 (46)
Mild neutropenia	2 (46)
Herpes simplex	1 (46)
Respiratory infection	1 (46)
Previous treatment	25 (46)
Methotrexate	5 (25)
Cyclosporin	2 (25)
Acitretin	17 (25)
Oral hormones	4 (25)
Etanercept and adalimumab	1 (25)
AnaKinra	1 (25)

## 4 Discussion

Generalized pustular psoriasis is a severe skin disease, potentially life-threatening, which is often accompanied by high fever, elevated white blood cells, and even sepsis (27). Current research has concluded that this disease is an independent disease that is significantly different from plaque psoriasis, both in terms of pathology and physiology (28). Immunosuppressants are usually the first-line therapy for acute inflammation, but their serious adverse effects and uncertain efficacy cause concern, their efficacy is slow and symptomatic improvement is inadequate (29). With the current in-depth research on psoriasis and the use of biological agents in its treatment (30–32), significant progress has been made, leading to good treatment efficacy (33, 34). A Japanese retrospective study of 1,516 cases of generalized pustular psoriasis showed that biologics have better efficacy compared to immunosoppressants (35). However, immunosoppressants are still used as the first-line treatment for pediatric and adolescent patients, they have definite efficacy in clinical treatment, but are very prone to relapse after stopping the drug (36). The efficacy and safety of biological agents in the clinic are not well defined. Secukinumab has demonstrated improved safety and efficacy in the early treatment of plaque psoriasis in children and adolescents (37). In a controlled study comparing secukinumab to acitretin for treating pustular psoriasis in adolescents, secukinumab was significantly more effective than acitretin in reducing fever, leukocyte elevation, and pustular regression, while also causing fewer adverse effects (23). This study summarizes the clinical efficacy of secukinumab in the treatment of generalized pustular psoriasis from 4 weeks to 45 weeks, with all patients achieving a GPPASI of 90 and above at 12 weeks, demonstrating the efficacy of secukinumab in the treatment of this disease. Therefore, it is expected to be a novel therapy for treating this disease in pediatric population. In acute GPP, the clinical symptoms are very severe, often characterized by high fever, muscle pain, and skin swelling. Early and adequate intervention is crucial. In the literature provided, there are reports of patients who experienced significant relief from fever, myalgia, and other symptoms in approximately 3 days (24). This indicates that secukinumab has a rapid onset of action. It also has better efficacy and safety in long-term maintenance therapy. However, considering the low level of evidence in the included literature, mostly case reports, more high-quality multicenter studies are needed to demonstrate the clinical efficacy and safety of secukinumab.

## 5 Conclusion

This study systematically evaluates the literature on the treatment of GPP in children and adolescents with secukinumab. The results indicate that secukinumab offers rapid symptomatic relief and demonstrates good clinical efficacy and safety in long-term follow-up. However, the conclusions need to be confirmed by more multi-center and large-sample clinical studies, taking into account the sample size and the quality of the literature.

## Author contributions

KW: Writing – original draft, Writing – review & editing. XH: Writing – original draft, Writing – review & editing. PL: Writing – review & editing. DY: Data curation, Formal analysis, Writing – original draft, Writing – review & editing. JL: Writing – original draft, Writing – review & editing. LL: Writing – original draft, Writing – review & editing. MX: Writing – original draft, Writing – review & editing.
